# The attachments of ‘autonomous’ vehicles

**DOI:** 10.1177/03063127211038752

**Published:** 2021-08-14

**Authors:** Chris Tennant, Jack Stilgoe

**Affiliations:** University College London, London, UK

**Keywords:** autonomous vehicles, attachments, technological narratives, responsible innovation, governance, self-driving vehicles

## Abstract

The ideal of the self-driving car replaces an error-prone human with an infallible, artificially intelligent driver. This narrative of autonomy promises liberation from the downsides of automobility, even if that means taking control away from autonomous, free-moving individuals. We look behind this narrative to understand the attachments that so-called ‘autonomous’ vehicles (AVs) are likely to have to the world. Drawing on 50 interviews with AV developers, researchers and other stakeholders, we explore the social and technological attachments that stakeholders see inside the vehicle, on the road and with the wider world. These range from software and hardware to the behaviours of other road users and the material, social and economic infrastructure that supports driving and self-driving. We describe how innovators understand, engage with or seek to escape from these attachments in three categories: ‘brute force’, which sees attachments as problems to be solved with more data, ‘solve the world one place at a time’, which sees attachments as limits on the technology’s reach and ‘reduce the complexity of the space’, which sees attachments as solutions to the problems encountered by technology developers. Understanding attachments provides a powerful way to anticipate various possible constitutions for the technology.

## Introduction

### The narrative of autonomy

In 2005, competitors in the second DARPA Grand Challenge designed driverless vehicles to navigate unfriendly desert terrain. Sebastian Thrun (2011a), whose team won the competition, explained how his team took a car, made it learn and ‘set it free in the desert’. Thrun (2011b) went on to found Google’s self-driving car project, where he evangelized about ‘a future without traffic accidents or congestion. A future where everyone can use a car’. The simple idea was that giant leaps in artificial intelligence would enable computers, sensing the world around them, to take over the task of driving from humans. One of our interviewees, who had taken part in the DARPA Challenge, explained how the competition had created a philosophy of robotic autonomy that shaped the development of self-driving cars:

You don’t get to do anything with the infrastructure. You have to … put enough intelligence in that one vehicle to get it to not crash in the existing environment and then just replicate … You could put all your billions into one car, get that to work … and the replication costs are essentially zero. That’s the brilliance. (Interviewee 16)

For Thrun and other enthusiasts, the promise of the autonomous, self-driving vehicle (AV) is that it will change the world without the world needing to change. The history of technology suggests this is unlikely. Technological promises, if they succeed, end up making demands on the world (Van Lente and Bakker, 2010). Autonomous vehicles are not as self-contained or self-governing as their proponents maintain (Stilgoe, 2018). To understand the opportunities and uncertainties of these innovations, we need to understand, first, how and why their developers publicly assert the technology’s autonomy, second, how these groups privately admit the many ways in which their vehicles are attached and enmeshed in social and technological complexities and, third, how they handle various attachments as they seek to make the technology work.

At the time of writing, early 2021, the technology has in a sense been set free. At least in the US, there are few formal rules governing its development. And yet it remains, for the time being, stuck in the desert. In Chandler, a suburb of Phoenix, Arizona, Waymo, the successor to Google’s self-driving car project, runs a fleet of cars without drivers on most of the roads within a 50 square mile area. The technology, in this place, for a small set of users and with a large cast of behind-the-scenes support staff, works, but it remains corralled.

Other companies, even if their technology is less developed, promise wider horizons. At an ‘Autonomy Day’ in 2019, Tesla CEO Elon Musk told investors: ‘I feel very confident predicting autonomous robotaxis for Tesla next year’, and promised that software updates to make this happen were imminent (Tesla, 2019). The driver-assistance system that the company formerly labelled ‘Autopilot’ has since 2016 been offered as a ‘Full Self-Driving’ upgrade. In 2015, Musk had told a journalist that the company would achieve ‘complete autonomy in approximately 2 years’ (Korosec, 2015).

Tesla is an extreme example, and Musk is a unique vanguard, but the narrative of autonomy is shared by almost all of the actors currently jostling for attention in what they see as a race to develop self-driving cars. Waymo claims that it is building ‘the world’s most experienced driver’ (Waymo Team, 2019), with an ‘ultimate goal to develop fully self-driving technology that can take someone from A to B, anytime, anywhere and in all conditions’ (Waymo, 2020).^1^ In 2021, the company changed the slogan of its public relations campaign from ‘Let’s talk self-driving’ to ‘Let’s talk autonomous driving’ (Waymo, 2021). Oxbotica (2020), a British AV company, has a plan to bring ‘autonomy to all vehicles, in all places at all times’. The narrative of autonomy has its own scripts and schematics. Typically, developers reference the SAE (Society of Automobile Engineers) levels of automation that climax with Level 5 ‘full autonomy’, a vehicle that ‘can drive everywhere in all conditions’ (SAE International, 2018).^2^ Taken at face value this implies complete independence from human operators, physical infrastructure and conditions, external digital infrastructures and other road users.

### Attachments

The narrative of autonomy sees AVs as detached. Other potential narratives acknowledge how the technology of autonomous vehicles is attached in many ways to the world around it. Bruno Latour (2011) argues that the modernist spirit of innovation – a ‘cult of autonomy’ (Hennion, 2017) – promises emancipation from attachments, while in fact creating more attachments. For Latour, attachments are obligations: relationships with people, objects, institutions and infrastructures that give a thing or person definition. Describing how emerging technologies are more attached than some of their developers like to claim is important if we want to develop more responsible modes of innovation (Stilgoe et al., 2013). We should therefore seek to understand the possible politics of technology through closer investigation of the attachments that developers anticipate and also those that they neglect or deny. Our paper is inspired by, but not organized within the more developed frameworks of, relational ideas of technological change such as actor-network theory. Our desire to explain the myriad possible relationships a nascent technology might have with the rest of the world means a looser sense of the term ‘attachment’ than that suggested by the actor-network theorists (Hennion, 2017). Latour’s (1996) study of Aramis, a French automated system, remains an obligatory passage point for any project addressing automated vehicle attachments, even though the technology he investigates pre-dates recent excitement about AI for self-driving cars.

We use the concept of technological ‘narratives’, following Sovacool and Hess (2017), who reviewed the many theories that address attempts to build discourses, imaginaries, visions and expectations of technological futures. Setting aside the distinctions theorists make between these different forms of meaning-making, we treat the narrative broadly, as a way for stakeholders to organize emerging technology discourses, contain contradictions, attract resources and embed preferred technologies within wider socio-technical systems.

Our paper uses interview data to understand the complexities and other potential narratives behind the distilled, detached autonomy described above. We draw on over 50 interviews with people involved in the technology. Sixteen interviewees worked in research and development at AV technology companies, a further eleven were technologists in academic positions, while six were from car companies. The rest were academic researchers, policymakers and other stakeholders. Twenty-two were from North America and 27 were from Europe. Interviews were conducted between 2019 and 2021 on condition of anonymity, and had an average length of around an hour. The topic guide began with interviewees’ expectations for AV technologies, before probing their sense of AVs’ potential relationships with their users and with other technologies, road users, infrastructures, policymakers and the general public. All interviewees are anonymized. As the technology is still young and categories have not yet stabilized, some interviewees play multiple roles – for example researcher and technology developer. Where appropriate and possible without jeopardizing anonymity, we give an interviewee’s background, discipline or sector. Our interview data is supplemented with insights gained from AV policy meetings, technology conferences and technology demonstrations in the UK and the US.

Using our interview material, we analyse how stakeholders describe the attachments that so-called ‘autonomous’ technologies have to the world. We consider how they make sense of the challenges faced in dealing with these attachments, how they construct strategies for understanding or downplaying them and how they internalize or externalize responsibility for their care. Finally, we consider how different views of attachments suggest governance alternatives for AVs.

## Autonomy, freedom and control

If autonomy is about freedom, agency and self-direction, the narrative of autonomy for AVs blends three senses of autonomy without acknowledging the potential clashes between them. First is the autonomy of the travelling individual, given new opportunities and freed from an imagined burden of driving. Second, there is the autonomy of the vehicle itself, powered by an artificial intelligence that is expected to mimic and then master the task of driving; the human driver is removed and replaced by a computer. The third idea is that of autonomous technology, in the sense described by Winner (1977). Technology is seen as having a life of its own, ‘out of control and … independent of human direction’ (Winner, 1977: 13). This positions the narrative within a master narrative of technological progress (Felt et al., 2007), with the normative implication that innovators should be unconstrained by regulators or societal concerns. This progressive ideal is bolstered by confidence that technology is inherently emancipatory. Even a cursory test of these narratives of autonomy shows that one’s view of autonomy and attachments depends on one’s standpoint. To an AV developer, a driver asserting her right to drive could seem reactionary, clinging to the outdated attachments of car culture, while a sceptical motorist might fear becoming dependent on tech companies if she wants to get from A to B.

### Autonomous travellers

Driverless vehicles promise an emancipatory technological sublime (Hildebrand, 2019): inclusion and mobility for those currently unable to drive and freedom from the downsides of today’s cars for those already driving. Yet the car itself was, and is, sold as an instrument of personal autonomy (Lomasky, 1997). Advertising – often depicting empty roads – has consolidated the ideal of freedom and self-expression for drivers (Dittmar, 1992; Stokes and Hallett, 1992). The automobile – literally ‘self-moving’ – has allowed drivers to project their egos far and wide, with the car and the driver fused into a single hybrid agent (Böhm et al., 2006; Dant, 2004).

Although advertisements for cars rarely show traffic, it is a central concern for human drivers. More broadly, drivers are enmeshed in a system of automobility (Urry, 2004) that offers opportunities while requiring relationships with those who make and sell cars, build and maintain roads, refine and distribute fuel and of course share the road. The car facilitates independence even as we are constrained by our dependence on it. Automobility envelops its users in a thick web of rules (Merriman, 2006; Seo, 2019; Vinsel, 2019). The driver is both liberated and regulated. This ambivalence also characterizes perspectives on the whole system: Congestion can be seen as evidence of a thriving society (Downs, 2004; Taylor, 2002) or as evidence of a poor allocation of road space (Roughgarden, 2016), with negative influence on quality of life (Steg and Gärling, 2007). Many people remain excluded from the economic and social flourishing that relies upon the system’s affordances.

Freeing the travelling public from the task of driving necessitates cleaving the hybrid of the driver-car (Dant, 2004). For upstart tech companies, the economic imperative is to break the ties that currently bind consumers into an analogue sociotechnical system of automobility (Urry, 2004) that is dominated by legacy car manufacturers and governed by rules that favour incumbents.

We should reflect upon the nature of the human agency that autonomy purports to mimic or hopes to replace. Social psychologist Kurt Lewin (1997 [1940]) proposed that an agent’s behaviour can be theorized as a function of its characteristics and its environment. Psychological accounts of agency stress the contextually cued automaticity of human behaviour (Bargh and Chartrand, 1999; Gigerenzer, 2008). When developers promise to learn from, replicate and then surpass human driving, they overlook the attachments that define the agency of a human driver. Given the constraints and conditions of human agency revealed by automobility, we should ask whether automated systems are likely to enable new freedoms or become similarly stuck in traffic.

### Heteronomous vehicles

We can learn from the real-world application of other systems whose designers make claims to autonomy. Suchman and Weber (2016) analyse definitions of autonomy for drone strike aircraft. While governments want to offer reassurance that there is always a human in control, autonomous weapons operate in ‘an open horizon of potentially relevant circumstances’ (Suchman and Weber, 2016: 92). Autonomy is therefore *‘*configured as self-sufficient, adaptive and self-determined performance on the one hand, and pre-programmed, fully automated execution under perfect human control on the other’ (Suchman and Weber, 2016: 90).

‘Autonomous’ machines have been programmed and deployed by humans, projecting the agency of their authors into the world (Mindell, 2015). Mindell challenges what he calls the ‘myth of replacement’ by arguing that technologies displace rather than replace humans – for example, to the control booth overseeing a submersible or a Mars rover. Mindell argues that full autonomy is a myth, that it is actually just ‘human action removed in time’ (Mindell, 2015: 220). When humans disappear from cars’ driving seats, we should therefore look carefully to see where else in the system, however broadly we can draw it, they might be working.^3^

That autonomous systems import human agency into their operational domains reminds us how heteronomous (governed by, and dependent upon, others rather than themselves) they are. Benjamin (2013) reports that it takes 168 people to keep an ‘unmanned’ Predator drone in the sky for 24 hours. A robot vacuum cleaner needs its operational domain tidied up by the owner (Royakkers and Van Est, 2015).

Human-machine teaming offers new opportunities (Van der Vecht et al., 2018), but the new social relations can blur responsibility and be exploitative. Human operators within automated systems often carry the blame for errors (Bainbridge, 1983; Elish, 2019) much as car companies blamed drivers for safety failings in the 1950s and 1960s (Nader, 1973) and more than 90% of automobile accidents are still blamed on driver error (Department for Transport, 2015). On closer inspection, the idea of autonomy operating here is one in which machines are emancipated from the constraints and complications of human operators.^4^

Suchman and Weber (2016) argue for ‘a shift in conceptions of agency and autonomy, from attributes inherent in entities, to effects of discourses and material practices that variously conjoin and/or delineate differences between humans and machines’ (p. 76). Melissa Cefkin, an anthropologist formerly at Nissan and now at Waymo, applies a similar approach to AVs:

Autonomy is not an attribute intrinsic to a single entity. Autonomy is rendered to the connections among and between people and objects, connections that give rise to action. It’s our actions that are autonomous, our abilities to act, and not the things themselves. So designing human machine interactions for the future means designing for our relational world. (Cefkin, 2017: at 44 minutes)

Seeing agency as relational is crucial if we want to see past, and improve upon, the narrative of autonomy. Drones, self-driving cars and other systems often labelled ‘autonomous’ could propagate human agency in a manner different from the physical barriers or technologically-enabled routines with which existing technologies have embodied or entrenched social relations. As the stakes of artificial intelligence become clearer, researchers are highlighting how machine autonomy could impact upon human autonomy and justice in profound ways (e.g. Eubanks, 2018; Winfield and Jirotka, 2018).

To anticipate the possible social constitutions of AVs, we need to understand the technology’s potential attachments. The narrative of autonomy seeks to escape from most of these complexities, attempting to deny the demands placed on the social and material world. We are interested in what it would mean to care for rather than neglect attachments (Puig de la Bellacasa, 2010). The next sections report on our interviews, in which we asked those involved in the technology to address the ways in which AVs might not be autonomous.

## Attachments in the car, on the road and with the wider world

When prompted, our interviewees recognized a set of attachments – social and technical, tangible and intangible – that demand their private, if not explicit, attention. In this section, we trace these attachments and consider why some get prioritized while others are downplayed. Our approach here is relational, highlighting issues rather than particular objects or stakeholders, because the nascent status of the technology means that the relevant actors are not well-defined. We consider three layers of attachments: in the car, on the road and with the wider world.^5^

### In the car

There are [AV] companies out there that say, well, ‘machine learning is the thing to do’ … But as a machine learning researcher I think, like, a naïve version of that is nonsense. (18)

Excitement about machine learning has led some to configure AV innovation as a pure AI problem, in which a computer can learn to drive using simple inputs and data on driver behaviour, without a model of the world and, as one AI researcher put it, ‘without a teacher’ (28). In the AV business this is sometimes called ‘end to end machine learning’ (Bojarski et al., 2016) or ‘pixels to pedals’, but few people who have spent time involved in R&D go that far. One interviewee, a former AV company employee, said:

If I was a very naïve investor, I would just look at it and be like, yeah, this totally makes sense. Like, you put in all the data and humans were basically just one deep learning system, and we know how to drive, so therefore a car should be able to do the same thing. The reality is much more difficult. (51)

All interviewees admitted that AVs cannot be purely autodidactic. They must also be taught some rules. These rules may include the formal rules of the road, but they may also include typical (and possibly deviant) driving behaviours that humans learn by doing. Rather than an autonomous, independent machine, a successful AV system collectivizes intelligence in the software that supports the whole fleet, ‘the back-end’:

The first point is that the vehicle does not learn. No vehicle learns. … a vehicle performs. It’s not learning. It’s only recording data. The data are sent to the back end… You learn in the back end. And you use, of course, artificial intelligence much more in the back end than you do inside the car. (27)

With its emphasis on AI, the narrative of autonomy foregrounds software. However, interviewees discussed contingencies of hardware, including maintenance, batteries and, most importantly, sensors. Most developers use Lidar^6^ for high-definition perception of the environment. Elon Musk has called Lidar a ‘crutch’; Tesla relies on computer vision using cheaper cameras. An AV industry consultant sought to downplay the sensor controversy:

Can Lidars make it happen faster? Most people say yes. He [Musk] says no … we’re waiting for a breakthrough really in computer vision [which would mean we could] use it without other sensors … Lidar is a crutch, but computer vision has one leg. So a crutch might be handy. (48)

There are also divergent views on what additional information an AV needs besides that generated by its own sensors. Most developers use high-definition mapping to ‘know somewhere before you can drive it’ (8) and compensate for unreliable GPS. The maps, generated from a car’s-eye rather than bird’s-eye view (although often then incorporated into existing top-down digital maps), are normally proprietary, and companies see their differences in approach as an important source of competitive advantage. The idea is that an AV should know, with centimetre precision, what fixed objects to expect. One tech company executive explained the problems of dependence this mapping of ‘priors’ might create:

You can’t bank on priors … You don’t have the … luxury of priors when it comes to dynamic objects, such as pedestrians. Priors are great when you have static things like roads, poles, traffic lights, but when it comes to cyclists and people and so on … you couldn’t tell that from a prior. (34)

Another put it more vividly:

[What if] the map diverges from the real world? Something the car sees doesn’t match up? Let’s say, it’s expecting a traffic light. And … there was… a windstorm and it like kind of cocked to the side. The car stops. The car just like freaks out and gets bricked. (51)

As with Lidar, Tesla (2019) says publicly that maps are a ‘crutch’, with Musk dismissing them as ‘an extremely bad idea: The system becomes extremely brittle’ (at 2:42:30). This ‘brittleness’ is mentioned by other developers, but Tesla has since admitted that they are using the data generated by their own vehicles to build maps, albeit without the detail afforded by Lidar. For Tesla, admitting dependence on maps means tying their vehicles to particular places, which would complicate their story of universal autonomy.

For now, the real crutch in AV systems is the driver. As of 2021, most AV companies running tests on public roads employ safety drivers and ride-along engineers, ready to do what the computer can’t yet manage and assessing the system’s performance. The inside joke is that you know a car is driverless because it’s got two people in the front. In the UK, most safety drivers involved in tests are highly-trained, in recognition of the knowledge that being in-the-loop or on-the-loop is more rather than less demanding than driving (see Goldenfein et al., 2019). It is notable that in cars sold with Advanced Driver Assistance Systems (ADAS) such as lane-keeping, automatic emergency braking and adaptive cruise control, drivers are often assuming responsibility for automation without training or clarity about these systems’ limits.

### On the road

Once we depart from the narrative of autonomy, in which computers are equated with humans in terms of their sensing and intelligence, we can consider how AVs will actually make sense of contexts and relationships on the road. Material infrastructure becomes vital, at a minimum to delineate the edges of the operational domain. Ernst Dickmanns, a German self-driving pioneer using machine vision in the 1980s (Dickmanns and Zapp, 1987), explained in an oral history interview how their system worked out the edge of the road:

It was white lines and dark-to-bright transitions at the side of the road. We didn’t have to have white, white lane markings on the road. But we could do it with a normal transition from macadam to just grass or whatever. (Dickmanns and Asaro, 2010)

One interviewee explained that because ‘the software learns on white lines’ (39) it can become dependent upon them, leading to vulnerabilities if the lines are obscured. When white lines are obscured, the system can fail, as happened when the mayor of Los Angeles was driven by a prototype Volvo; in this case, the Volvo team were quick to blame the infrastructure (Stilgoe, 2017).

Developers also debate whether the car should connect to other road users and surrounding infrastructure to supplement its own perception systems or allow its movements to be co-ordinated by traffic management systems. Some interviewees saw cybersecurity risks that justified autonomy rather than connectivity: ‘it’s kind of like the lesson you teach small children: Do not talk to strangers …. It’s a good lesson for cars, too’ (48). For others, particularly those with backgrounds in systems engineering, the possibilities of digital connectivity make the disconnectedness of human drivers seem inefficient and unsafe. Connectivity would allow AVs to ‘know what’s around the corner’ (9). Connections could be made between vehicles (‘V2V’) or with infrastructure (‘V2I’). One developer told us how, when assessing whether a traffic light was green or red, ‘Right now we just use computer vision’ (18), but admitted that this was suboptimal. In some city trials, AV developers have added transmitters that communicate traffic light status directly to the vehicle. And, as part of a new standard for Dedicated Short Range Communications, many new traffic lights are now built with the ability to communicate their location, phase (colour) and timing via radio. As 5G networks are built, AVs have become part of many countries’ policy justification.

Interactions between an AV and others on the road could be understood as a set of short-lived micro-attachments. However, from the AV’s point of view, pedestrians, cyclists and other vehicles are just more things to be perceived, anticipated and avoided. Developers talk about their car as an ‘ego vehicle’ and others as ‘target vehicles’ (33). Pedestrians become ‘dynamic objects’ (34), classified along with buses, cars and bikes in ‘bounding boxes’ (29) within the system’s model of the world so that their behaviour can be predicted. Interactions with the AV are assessed probabilistically according to cost and reward functions that balance safety with efficiency:

So the classification, you know, is that a pedestrian or not? … How many nines? [a measure of reliability: 99%, 99.99% certainty etc] That isn’t what you need. What you need is ‘Is that a pedestrian who’s about to step off the curb or is that a pedestrian who’s waiting for the bus?’ … You probably need that level of classification to be good at prediction. (36)

Developers need to demonstrate progress. Many post videos (often sped-up) of tests on public roads, offering a view through the windscreen, and often accompanied by schematic representation of the roadscape, including the ‘bounding boxes’ of object recognition and classification. These performances posted online are joined by amateur footage from users that shows both the successes and limits of the technology (for critical appraisal of user videos see Brown and Laurier, 2017).

### The wider world

The technological achievements of AVs will not speak for themselves. The technology depends upon public support, the continued exuberance of investors and benign conditions created by policymakers. Behind the curtain at the companies developing AVs, there is substantial hidden labour involved in preserving the narrative of autonomy. As well as the safety drivers overseeing AV tests, most systems depend upon countless acts of micro-work (Irani, 2013) such as data-labelling, test-driving, teleoperation and customer support (Ganesh, 2020), a process that Ekbia and Nardi (2014) call ‘heteromation’. Its costs are seen by most developers as a short-term investment, which will collapse once AVs are ready for use at scale, rather than a permanent fixture of systems.

All companies rely on data that has been painstakingly labelled. Most of us will by now have been enrolled, through Google’s Recaptcha system that purports to distinguish humans from bots, in the process of AV data-labelling, identifying features in (normally American) streetscapes. One AV mapping specialist told us about ‘a team of 100 in India to label these point clouds’ (51) that come from Lidar sensors in the development of HD maps. Where companies have removed drivers from test vehicles, they have started to use remote operators to intervene in complex situations or even support personnel in vehicles that follow the prototypes, ready to manoeuvre them out of tight spots. Tesla uses its consumers both for data-collection and for vicarious experimentation (Brown and Laurier, 2017).

Developers encourage investor expectations, and those expectations shape the business models that developers fit their technology to. The finances often seem fanciful (Nunes and Hernandez, 2020). Mostly, the economics are implied: The aim is to win a race, perpetuating the ‘Grand Challenge’ mentality of the DARPA desert race (Kaldewey, 2018). The goal, as some interviewees put it, is to ‘solve’ (48) or ‘crack autonomy’ (25), thereby capturing monopoly rents. The assumption is that, as with a new app, innovation will trickle down across readymade infrastructures, spreading benefits across places and user groups. The current workforce and hardware (a 2020 Waymo vehicle costs about $200,000) are seen as up-front investments rather than running costs. Some interviewees saw the finances only working if the technology approached universality and was therefore scaleable at ‘essentially zero’ (16) cost. Others presumed that the ‘race’, even if it didn’t reach some imagined finish line, would have many losers but the leaders would find some way to monetize the successes.

For tech developers, Silicon Valley economics are fundamental to their own operating environment, imposing the need to hit milestones to move through funding rounds and the demand for business model scaleability.^7^ This creates incentives to hype, as one university engineer described:

We have people in the industry who want to pump up the value of their companies, both big well-established companies and start-ups … one company goes out and makes an aggressive claim, and all the competitors have to make sure they’re not perceived to be left behind, so they’ve got to match it. (1)

Developers are keen to extrapolate the implications of their technology – imagining worlds in which nobody dies on the roads, commutes can be longer, suburbs can expand etc. They are less able to imagine the wider world speaking back, and less willing to imagine a mutual relationship with the wider world that might imply dependencies. One interviewee’s characterization of where the road meets the wider world is telling:

The problem is where it interfaces with the people …. The biggest infrastructure change is going to happen at the curb, when people get in and get out. (16)

Calling ‘interfaces with the people’ a problem is no mere slip of the tongue. Other road users introduce unscripted complexity for AV systems. One interviewee said, ‘there is nothing in the driver’s handbook about what to do if people cross the street on a skateboard’ (3). And developers typically see public opinion, law and regulation as barriers to the testing and deployment of their technology. One interviewee argued ‘society is the biggest barrier to widescale adoption and acceptance’ (21). Another saw regulators as not just unhelpful, but also self-interested:

If you’re a regulator or an auto regulator, is it ‘Oh, well, then I must play an important role in this, of course, and therefore, let’s start thinking how we regulate this before it even exists’. (48)

Early policy discussions have focussed on liability. Existing legal regimes presume the presence of a responsible driver (e.g. Law Commission and Scottish Law Commission, 2019). The need to attribute responsibility to a specific agent follows from imagining agency in individualistic rather than relational terms.^8^ Automotive companies have always blamed the driver for accidents (see 2.2 above), a default that one safety researcher termed ‘The driver error narrative’ (Koopman, 2018: 7). Without a driver in place, someone else, or something else, has to take responsibility.

Developers see inevitable attachments with public opinion, typically problematized in terms of public ‘acceptance’ (Tennant et al., 2019):

I mean, systems will never be 100% perfect, we cannot predict everything, so there will always be accidents. And that is probably a question of acceptance. (34)

On the question of how safe is safe enough for new technologies, one engineer responded, ‘I’m throwing the ball over to you’ (16), casting us, the social scientists, as calculators of public opinion. Another interviewee saw the challenge as being more than technological:

You’ve got to do it in a way that will sort of maximize that deployment. And that’s not just a technological achievement. It’s a business achievement. It’s a regulatory achievement. It’s a public acceptance achievement. (48)

From this view, the attachments to the wider world provide just another set of problems to be solved.

## Harder than first thought

An interviewee from a safety engineering background told us,

there’s a realization that it might be very difficult to deploy … outside of, you know, a very narrow, operational design domain such as, at the moment, Chandler in Arizona … it’s harder than first thought. (46)

Most of our interviews took place during a period of reckoning among AV developers in which they and the journalists covering them publicly backpedalled on their earlier hype (Economist, 2019; Piper, 2020). One engineer, who had participated in the DARPA Grand Challenge before moving to a start-up, reflected on how the excitement had waned:

It went from people being amazed that it was even possible to drive 100 miles without a driver in a car in this sort of DARPA test scenario to ‘Why don’t I have my self-driving car yet?’ (32)

Another said, ‘a lot of people underestimated the complexity of the problem’ (3). It is less clear exactly whose expectations – developers’, journalists’ or the public’s – were being dashed. Some developers have blamed the media for hype while others have been more reflexive.^9^

One developer working on standards as well as the technology saw a switch in 2018:

The industry’s public projections and policy positions and engagement with standards and policy conversation are not quite yet fully caught up to, I’d say, the post 2018 trajectory of autonomous vehicles. (35)

Another saw the dynamic in terms of technological naivete:

You’ve got a couple of guys with PhDs in robotics and they think they’re great and they say, ‘Right, let’s start a driverless car company because I’m sure we could sell it for billions. You go off and do the GPS because I’m sure we’ll need GPS and you go off and figure out HD maps and I’ll figure out how to follow white lines in the road, you know, we’ll do alright.’ So, these guys make incredibly rapid progress because they’re all smart people and within a couple of months they can show you a car on the road following a map using GPS and then throwing in white lines as well for those more difficult situations… and then they run out of runway because they haven’t got the fundamental technologies… they’re going to find it harder to work across all the seasons, they’ll find it harder when the road changes or roadworks happen. (8)

Although many interviewees refer to the complexity of ‘the problem’, our interviews revealed multiple framings of ‘the problem’, each of which conflates particular engineering challenges with social problems. As the more ambitious developers have come to terms with their own disappointments they have been forced to engage with attachments that had previously been neglected.

We saw three main ways in which developers made sense of their attachments and attempted to bring them under control, each of which blends different logical, technological and ideological motivations.

1. ‘Brute force’: attachments as problems.
2. ‘Solving the world one place at a time’: attachments as limits.
3. ‘Reduce the complexity’: attachments as solutions.

### ‘Brute force’: Attachments as problems

What a lot of people are trying to do is essentially brute force, and brute force works in chess. It’s an empirical question whether it will work in driving … If we get five orders of magnitude more data, if that’s even possible, maybe we’ll get, you know, five orders of magnitude increase in accuracy? (3)

‘Brute force’ here means relying on massive datasets rather than formal modelling of the rules of driving. Another engineer, who was similarly sceptical of this approach, described it like this:

Everyone’s like, yeah, we’ll just throw a bunch of data at it. Enough data to teach a human and then out the other end will come a human-, better than human-, level driver. (51)

Some AV developers nevertheless continue to claim, even in private, that, with enough computing resources (data, processing, programming expertise), they will be able to account for and internalize all of the complexities they encounter.

Asked whether the wider world would need to change to accommodate their tech, one developer replied, ‘We’ve never thought about encouraging adaption to any infrastructure or societal behaviour to help support our AV deployment’. When prompted to talk about small upgrades like smart traffic lights, the response was ‘we don’t really deal with connectivity. [The company] likes to solve very difficult tasks’ (33). Another told us that ‘Pedestrians don’t need to do anything different’ (47) around their vehicles.

This approach reflects a faith in machine learning and a conviction that the outside world will not or should not change. Some developers, particularly those in the US and those looking to move quickly, have no intention of seeking help from infrastructure. One tech executive explained:

On a venture capital time frame … we just can’t buy into that because we just think that it’s going to require way too much cooperation, coordination with the public sector and in the US anyway, we don’t do big infrastructure projects anymore… You don’t get to do anything with the infrastructure. You have to … put the intelligence in the vehicle (51).

Others see the issue as one of responsibility: ‘the idea that you would change the world to solve your problems is a crazy one’ (48). Asked about segregating roads to exclude other users from AV spaces, one developer said this would be, ‘running away from the problem instead of solving it’ (47). One technologist explained that dependence on high definition maps with ‘a lane graph that tells you where the lane boundaries are … just one giant graph with a lot of arrows … well, that kind of feels like cheating’ (51).

AV developers must constantly contend with the interface between their model and the world. Everyday encounters with previously unseen entities become, for the AV developer, ‘edge cases’. Some AV developers also mention how seriously they take ‘corner cases’ (13), events that push at the limits of two or more dimensions or edges of their model. Developers congratulate themselves on the identification and avoidance of outliers that, to a human, would be normally abnormal, even if they recognize that ‘the number of scenarios that we need to test against, even for relatively small ODD [operational design domain], is almost infinite’ (33). One interviewee, working on low-speed driverless shuttles, said that their vehicle’s response to edge cases was simple: stop and wait. Others saw the need when navigating an uncontrolled environment to balance absolute safety with nevertheless making progress. Some, particularly those involved in safety engineering, thought that bringing edge cases into the model was not straightforward. One engineer, discussing how humans drive in snow and other rare situations, concluded,

We don’t have a clue there how we’re gonna address that. The whole reason we need, like massive amounts of data is precisely because we don’t have a better understanding of common sense. (18)

Another saw edge cases as more than just data points, saying ‘you have to deal with them explicitly’ (7), by understanding what they are cases of. This demand for deeper modelling of scenarios, transparency and interpretability threatens to split AV developers. One AV programmer said ‘you shouldn’t care how I built the system’ (47) because what mattered was the validation of its performance. A safety engineer was critical of this black-box approach, defending his ‘huge database of edge cases with everything inside’ on the grounds that ‘a system which we bring to the road always needs to be 100% deterministic …. If you say, ‘Well, I don’t know what happened, there’s a deep neural network’, that won’t work’ (27).

One interviewee dismissed developers’ early efforts, saying ‘they wanted to drive like a human being does, which is just observe the environment around them and follow the white lines’ (25). As testing continues, edge cases proliferate and potential legal liabilities start to become clear, most developers now admit that AVs cannot just learn to drive through accumulating data. The more data is gathered, the clearer the limits of the technology become.

### ‘Solve the world one place at a time’: Attachments as limits

The strategy, though, is not one that says you drive everywhere in the world like Tesla maybe wants … You have to solve the world one place at a time. (48)

Unfamiliar places, especially those with haphazard driving cultures, could present an impenetrable forest of edge cases.^10^ Some developers saw the need to steer clear of the edge, staying within the boundaries of the familiar. One AV researcher concluded, ‘For the foreseeable future, they’re all going to be in a sandbox’ (56), an operational design domain (ODD) with controlled conditions to allow experimentation. SAE International (2018: Sect 3.22) defines an ODD as the ‘operating conditions under which a given driving automation system or feature thereof is specifically designed to function including, but not limited to, environmental, geographical and time-of-day restrictions and/or the requisite presence or absence of certain traffic or roadway characteristics’. In reality, many developers are not designing to tight specifications: They are exploring the conditionality of their technology by testing its edges. ODDs may then be defined in order to satisfy the expectations of others that there should be an explicit ODD rather than to provide transparency as to the technology’s capabilities. One developer told us their company had ‘a very well-defined definition of these ODDs. The details of it are a bit proprietary’ (18). The circumscription of a place is sometimes known as a ‘geofence’. Places can be mapped in detail, brought under control and demarcated from terra incognita, although the limited scope of a geofence is unlikely to be emphasized, because it admits limitations and troubles the story of autonomy.

This approach bounds AVs by location and other conditions. Some developers looking for early markets have sought to automate trucking and limit the ODD to predictable highways. But such a pragmatic approach may not go unpunished by investors who were originally persuaded by a more expansive narrative of autonomy. One start-up CEO who had targeted freeways as a relatively controlled ODD told us that investors ‘seemed to think I was admitting that my engineering team wasn’t good’ (29). Existing carmakers have invested in developing ADAS features such as lane-keeping and adaptive cruise control that give the impression of a self-driving car in predictable ODDs such as motorways.

To hear engineers talk about geofences and ODDs is to appreciate that the question is not when self-driving cars will arrive, but where. The conditions that constrain AV systems will define them. An ODD cannot just be represented by physical geography. The conditions for driving are not fixed to a particular space or weather pattern. A street may at different times be the setting for a traffic jam, a parade, a construction project or a crash site. Places, in all of their social richness, cannot be reduced to mere spaces (Gieryn, 2000) even if they are, like freeways or motorways, ‘reasonably scripted’ (18), as one AI researcher put it. The edges of an ODD, defined in technical terms, will not match the local knowledge of drivers or other citizens. One engineer argued,

The big technological challenge is now how to relax the constraints of the environment in which the vehicles operate, and enable them to start co-existing with other road users. (1)

Interviewees highlighted a range of constraints, including variability in the physical environment (e.g. visibility in difficult weather; readability of road markings and signs), predictability of other road users (such as the legibility of pedestrian intent) and the reliability and adequacy of external technological input (such as maps or V2I). In seeking to colonize further areas, the pressure is therefore to make the world fit the ODD rather than vice versa.

### ‘Reduce the complexity of the space’: Attachments as solutions

You could spend all your life solving every encounter and every use case, but you can’t have full coverage…. How do I minimize an infinite number of use cases? I reduce the complexity of the space… That’s the only way to go after this. (50)

Some developers take a pragmatic view, recognizing the limits of ever-expanding ODDs and proliferating edge cases, and conclude that the world around the AV could and should play a role in the technology’s success. One machine learning researcher said,

It’s unlikely that the machine will be able to totally adapt to us… How can we adapt our existing transport infrastructure to accommodate new modes of transport based on semi-autonomous vehicles or autonomous vehicles that are limited in scope? (23)

These interviewees see the rationale for what Law (1987) calls ‘heterogenous engineering’, and they recognize that it means taking control away from AV developers alone. Some saw infrastructure as far from peripheral.^11^

The virtue of a geofence is, you can throw large amounts but finite amounts of money at fixing infrastructure problems …. You can basically make it a semi-controlled environment if it’s geofenced. (55)

An AV policy lead at a large carmaker predicted:

infrastructure will change. Definitely. … And also it needs to change actively and not passively as it has done in the past … infrastructure was always behind. Now it has the opportunity almost to jump ahead … in China, for example, they – okay, completely different system there – they are really taking the lead on that. (54)

This interviewee went on to discuss the rules of the road:

we’re sort of playing within the borders of existing traffic rules. … we have the opportunity to maybe change them. … How much of the responsibility do you put on to the people, the public, and how much responsibility do you put on the manufacturer, the people who own the cars, the cars themselves, etc? I think you’re gonna see a change in social contract, potentially, there. And at the same time, I think having no responsibility whatsoever on the public is unrealistic. I think there needs to be education and training. (54)

One AV developer stopped short of claiming new infrastructure was required, but argued that it would help:

What we can do is try to help educate what the limitations are on ways that, if cities and if customers want this technology to become more widespread, these are ways that we could accelerate that effectively… infrastructure upgrades that just generally make transportation in cities safer are the kinds of things that are good for making self-driving more viable too. (32)

The infrastructure upgrades this interviewee mentioned included segregated bike lanes and protected pedestrian crossings, both of which are contentious for urban planning. Conversations about infrastructure tended to prompt a retreat to established forms of mobility:

I mean, you can take a subset of the problem and make it well constrained. We have had that for years. We call those monorails. (3)

AVs in some sense are just trains. And if you have a fixed route, that’s the train track, it’s kind of a logical train track, but a train track nonetheless. (52)

Other interviewees mentioned hard-to-model pedestrians as a particular concern. One academic researcher took a strong line: ‘Self-driving is something which I see in a controlled scenario essentially to solve the problem. … controlled scenario needs to be one where pedestrians are not allowed’ (39). An AV researcher said his job would be made much easier ‘If people could agree to cross at zebra crossings’ (18) echoing efforts to discipline pedestrians in the 1920s (Norton, 2008). An academic machine learning specialist suggested a similar approach:

the sort of authoritarian version is you outlaw jaywalking in order to reduce the problem. … I mean, it might work in China. It’s probably not going to work in the United States. And it’s also a reminder, if you think about it, of how dumb the machines are. It’s like we can only get the machines to work if we do some pretty drastic things to people. (3)

This researcher’s prognosis was based on a diagnosis that AVs could never deal with Suchman and Weber (2016: 92)’s ‘open horizon of possible relevant circumstances’:

We don’t have a full handle on all of the possibilities that arise in the real world… because the world is combinatorial and new conjunctions of things can happen. It’s not clear you can have an exhaustive list. So, um, you know, maybe you’ve never had a hawk and a violin fall off of a truck at the same time and, you know, maybe you need to decide what to do about it. You’re not going to have data on that. (3)

As part of a discussion about whether AVs should be clearly labelled for pedestrians and other road users, or whether they should carry ‘external interfaces’ (26) to communicate their intentions, one developer was concerned that this ‘forces [pedestrians] to become responsible parties’ (26), but suggested a mutual relationship was both honest and pragmatically attractive. A reciprocal relationship would seek to make a virtue out of attachments.

The narrative of autonomy sees the public as a problem: as bad drivers to be replaced, pedestrians whose movements must either be predicted or constrained, or citizens with an irrational scepticism of technology. Some interviewees focussed on on-road behaviour: ‘the biggest barrier to driverless cars is drivers … because the way that human beings behave at the moment is … it’s just, you know, they’re unpredictable’ (11). One argued, ‘are we going to have to tell people not to walk out in front of cars? I think we might’ (21). The implication is that changing the world for the better would require ‘educating the public’ (50). One engineer said that the logic of self-driving would make this happen anyway:

These systems educate people … the car will not let you overtake in a reckless manner… you try to accelerate too fast and it will not let you … people will adopt the driving style which will be more safe. (47)

Only a few interviewees were comfortable, even in private, discussing the ways in which we might need to, in the words of one, ‘organize the world differently’ (50) to accommodate their AVs. Some respondents had thought deeply and pragmatically about the trade-offs that society might have to consider in a self-driving future. Navigating these choices will require a wider view of policy and design (Blyth et al., 2016) than the narrative of autonomy currently allows.

## Reconnecting the autonomous vehicle

Investment in and excitement about autonomous vehicles is already starting to materialize in systems that operate in constrained ways in some places, but the narrative of autonomy that supported early enthusiasm looks less stable than it once did. Waymo admits that ‘autonomy will always have some constraints’, even as their publicity encourages belief in autonomous driving ‘anywhere, anytime, in all conditions’ (Tibken, 2018). The narrative that points to detached autonomy as the goal postpones public consideration of the technology’s attachments. The sequence goes: first solve the technical problem (get the artificial intelligence to work), then think about the rest. But the technology will not ‘work’ without considering its attachments. And, behind the scenes, developers are frantically seeking to make sense of the attachments that they privately know will define their futures.

Where developers acknowledge society’s role, they look for help to get the technology introduced: Policymakers are called upon to resolve issues in preparation for the introduction of new agents to the road, whether it be their legal status or their moral standing: Who is responsible when an AV crashes? (Brodsky, 2016)? How should an AV make value judgements in the event of unavoidable deaths? (Awad et al., 2018; and for a critique see JafariNaimi, 2017). Successful introduction is seen as requiring societal agreement upon criteria for risk assessment (McDermid et al., 2019) and definitions to decide on adequate safety (Marchant and Lindor, 2012).

These governance questions follow, rather than challenge, the narrative of autonomy. This is a form of AI reductionism (see Krimsky, 2005 for a characterization of genetic reductionism in biotechnology governance), which we might call autonomism,^12^ in which the issues are seen as flowing from artificial intelligence: Designers are focussed on getting the technology to work in narrow terms rather than on systemic concerns. Autonomism blends engineering practicalities with business needs and ideological commitments. ‘Autonomists’ expect governance to facilitate their innovations (Tennant et al., in press). Where questions of inequality figure, they focus on employment, such as the potential impact of self-driving trucks on drivers (Gittleman and Monaco, 2019). Autonomism seeks to escape from the societal discussion of either the means – how attachments might help the technology to work – or ends of AV technology.

As AVs move through an open-ended world they depend on their attachments, imposing obligations on a supporting cast of other actors. Scrutinizing the attachments of ‘autonomous’ vehicles and their developers’ own understandings of these attachments is a powerful way to challenge a technologically determinist view that takes the problem (human error) and the solution (artificial intelligence) for granted. Acknowledging the attachments would lay the foundations for a more inclusive constitution of autonomous vehicles, one that makes the introduction of the technology a means to societal goals (safety, sustainability, accessible mobility, etc.) rather than an end in itself.

In Latour’s (1996) autopsy of Aramis, a French proto-AV system, he reports that its developers ‘believed in the autonomy of technology’ so strongly that they ‘left Aramis to cope under its own steam when it was actually weak and fragile’ (p. 292). However, because the system was actually inextricably attached, and needed the world to compensate for its limits, the criteria with which to explain its failure are not obvious. The successes or failures of self-driving vehicles, located where the technology meets the world, will be similarly hard to calculate.

The narrative of autonomy for AVs looks increasingly unstable, but most AV developers find themselves stuck in their story, fulfilling Winner’s (1977) vision of ‘technics-out-of-control’. The promise, and the attention and money that follow, depend on an ideal of a technology that can scale rapidly and cheaply (Pfotenhauer et al., in press). It remains to be seen how forceful this narrative is (Van Lente, 2000). It could colonize and reshape a range of attachments, its failure could result in a retreat and a rethink, or it could lead to compromises from both innovators and others. It’s clear, however, that if AV developers and policymakers neglect attachments upstream, they will make demands upon the attachments downstream. AVs need not work on their own terms in order to change the world. By the time the limits of the narrative of detached autonomy become clear, commitments to the technology could be so entrenched that other parts of the system are forced to compensate. Infrastructures could be made to be more machine-readable, pedestrians could be made to be more predictable, roads could be reshaped to be more navigable, other road users could be expected to be connected at all times, drivers could be expected to take responsibility for constant oversight of partially automated systems, and sceptical members of the public could be asked to accept risks and other injustices as the price of overall improvements in average safety. A temporary apparatus of devices and social conditions currently supports AV trials: safety drivers behind the wheel, public roads as test sites, dedicated infrastructures, but also deregulation, exculpation and financialization. This situation grants AV developers temporary freedom from having to consider their attachments, but parts of it could become permanent, forcing the world to adapt to AVs rather than the other way round. Places with complex roadscapes and crumbling infrastructure could be encouraged to adjust and repair in order to reap the benefits of AVs. Attributes of places such as diversity, vitality and complexity that many people value but are hard to measure (Glaeser, 2000) might suffer from a drive for homogeneous efficiency. Devices, from Lidar systems to HD maps, currently seen by some developers as ‘crutches’, could become necessities. Rather than AV developers ‘solving the world one place at a time’, the world might aquiesce one place at a time. There are myriad ways in which the promise of autonomy could become a set of demands (see Van Lente and Bakker, 2010).

The more profound ethics of AVs will therefore come not from their supposed autonomy, but from their attachments. What would it mean to take greater care of those ‘neglected things’ in the material and social world to which AVs are attached (Puig de la Bellacasa, 2010)? The literature on mobility justice (Sheller, 2018) offers help. Worlds are shaped and reshaped around the affordances and dependencies of mobility technologies. The question is not when AVs will arrive or how to accelerate that arrival. Instead, we should be asking where the technology would be appropriate, who is likely to benefit, what form it could take and what ends should be prioritized. Rather than hoping for emancipation, we should ask what it would take for AVs to counter rather than exacerbate existing inequalities.

Early signs of a geopolitics of AV attachments are already starting to emerge. Our US interviewees admitted that the pragmatic driver of their autonomy is that the US appears to have given up on infrastructural progress (‘infrastructure changes so slowly’, in the words of one). This fuels a brute force approach to AI and a focus on places – Arizona, for example, rather than Manhattan – where simpler attachments make for easier conditions. The cultural differences between Silicon Valley and Detroit can also be explained in terms of alternative attachment strategies. Although the incumbent Detroit carmakers claim to be as excited about automation as the tech upstarts in Silicon Valley, they remain deeply attached to their current customer, the autonomous driver. Meanwhile, policymakers and AV developers in Europe, China and elsewhere are proposing standards for new infrastructure, vehicle-to-vehicle communication and upgrades to the rules of the road. Communication networks based on 5G (already the subject of international sabre-rattling) or alternatives, could either standardize or cleave AV system developments in different places. In China, where the separation between private and public sector actors is less clear, some places are already being designed around imagined AVs so that the technology will be easier to attach. Just as Aramis was a ‘unique [assertion] of French national identity’ (Edwards, 1997) so we can expect AV systems to look very different, textured by their attachments, in different places.

Superficial accounts of self-driving cars suggest a ‘race’ to ‘solve’ a singular ‘autonomy’. Behind the scenes, AV developers have different aims, emphasizing different attachments and targeting different niches. Alongside the possibility of consolidation to a few dominant players, there will also be differentiation as AV companies jostle to attach themselves to the world for competitive advantage. Optimistically, we might conclude that the social constitution of the autonomous vehicle is not yet set. But it remains to be seen whether the technology’s developers will break free from the narrative of autonomy or further entangle society within its effects.

## Notes

1 In 2018 the Waymo CEO admitted that ‘Autonomy will always have some constraints’ (Tibken, 2018).

2 The engineering standards organization SAE International (2016) defines six levels of automation from Level (L) 0 to L5. An L4 system can be operated in driverless mode in defined but limited conditions. An L2 system requires continuous monitoring by the driver. All systems need to have a defined operational design domain (ODD) specifying where an in what conditions it can operate (see Shladover, 2018 for critical analysis).

3 See Stayton (2020) for an excellent ethnographic analysis of engineers’ attempts to make automated cars drive like humans.

4 This idea is well illustrated by Kurt Vonnegut (1952/1985) in his novel Player Piano: ‘“If only it weren’t for the people, the goddamned people”, said Finnerty, “always getting tangled up in the machinery. If it weren’t for them, earth would be an engineer’s paradise.”’

5 Building neat typologies of attachments is hard. Starting with an object such as a car immediately invites consideration of the road. The structure here is a modest attempt to improve upon what Bogost, perhaps unfairly, characterizes as ‘Latour litanies’ of relevant things (see Seaver, 2017).

6 Lidar stands for Light Detection and Ranging. In AVs lasers can be reflected back of objects and measuring the time taken enables the location of the object to be defined.

7 These same pressures apply to the incumbent automakers. They too need to meet development milestones to convince shareholders that their AV activities can compete with Silicon Valley, even as they are constrained by competing shareholder demands for profitability as a mature technology.

8 This is also true of the individual agency ascribed to ‘moral machines’ (Awad et al., 2018).

9 Karl Iagnemma, founder of nuTonomy, admitted: ‘Vehicles are these massively complex systems, and to [build self-driving cars], we need to integrate them with another very complex system and do it in a way that’s reliable and cost-optimized. It’s really, really hard … I think that’s one of the things that most players in the industry underappreciated, myself included’ (Barnden, 2020)

10 Such frontier challenges were anticipated by Collins (1990) who argued in his early work on AI that the machine’s ‘intelligence’ depends on the extent to which an environment can be digitized.

11 There are echoes here of the prehistory of self-driving, when roads were seen as control devices (Wetmore, 2020). One early researcher from RCA explained ‘my idea was that control of automobiles should be done by the road’ (Zworykin and Heyer, 1975). Bel Geddes (1940/2017) explained the vision behind the 1939 General Motors Futurama exhibit at the World’s Fair: ‘Everything will be designed by engineering, not by legislation. … The two, the car and the road, are both essential to the realization of automatic safety. It is a job that must be done by motor-car manufacturers and road builders cooperatively’.

12 Townsend (2020) has coined the term ‘autonomists’ to describe technologists with both confidence in the power of AI for self-driving and an aversion to external regulation.

## References

[bibr1-03063127211038752] Awad E Dsouza S Kim R , et al. (2018) The moral machine experiment. Nature 563(7729): 59–64. 30356211 10.1038/s41586-018-0637-6

[bibr2-03063127211038752] Bainbridge L (1983) Ironies of automation. Automatica 19(6): 775–779.

[bibr3-03063127211038752] Bargh JA Chartrand TL (1999) The unbearable automaticity of being. American Psychologist 54(7): 462–479.

[bibr4-03063127211038752] Barnden C (2020) Watches, hedge funds and AVs: Complex or complicated? EE Times, 7 October. Available at: https://www.eetimes.com/watches-hedge-funds-and-avs-complex-or-complicated/ (accessed 23 July 2021).

[bibr5-03063127211038752] Bel Geddes N (1940/2017) Magic Motorways. London: Forgotten Books.

[bibr6-03063127211038752] Benjamin M (2013) Drone Warfare: Killing by Remote Control. London: Verso Books.

[bibr7-03063127211038752] Blyth P-L Mladenović MN Nardi B , et al. (2016) Expanding the design horizon for self-driving vehicles: Distributing benefits and burdens. IEEE Technology and Society Magazine 35(3): 44–49.

[bibr8-03063127211038752] Böhm S Jones C Land C , et al. (2006) Conceptualizing automobility: Introduction: Impossibilities of automobility. The Sociological Review 54(Suppl. 1): 3–16.

[bibr9-03063127211038752] Bojarski M Testa DD Dworakowski D , et al. (2016) End to end learning for self-driving cars. arXiv: 1604.07316.

[bibr10-03063127211038752] Brodsky JS (2016) Autonomous vehicle regulation: How an uncertain legal landscape may hit the brakes on self-driving cars. Berkeley Technology Law Journal 31(2): 851–878.

[bibr11-03063127211038752] Brown B Laurier E (2017) The trouble with autopilots: Assisted and autonomous driving on the social road. In: Proceedings of the 2017 CHI conference on human factors in computing systems, Denver, Colorado, USA, May 2017, 416–429. New York, NY: Association for Computing Machinery.

[bibr12-03063127211038752] Cefkin M (2017) Walk, Don’t Walk: Everyday Interactions with Self-Driving Cars. Available at: https://www.youtube.com/watch?v=m7ZFE3U_VY0 (accessed 12 July 2020).

[bibr13-03063127211038752] Collins HM (1990) Artificial Experts: Social Knowledge and Intelligent Machines. Cambridge, MA: MIT Press.

[bibr14-03063127211038752] Dant T (2004) The driver-car. Theory, Culture & Society 21(4–5): 61–79.

[bibr15-03063127211038752] Department for Transport (2015) The pathway to driverless cars: A detailed review of regulations for automated vehicle technologies. Available at: https://www.gov.uk/government/publications/driverless-cars-in-the-uk-a-regulatory-review (accessed 23 July, 2021).

[bibr16-03063127211038752] Dickmanns ED Asaro P (2010) Oral-History: Ernst Dickmanns. Available at: https://ethw.org/Oral-History:Ernst_Dickmanns (accessed 15 November 2020).

[bibr17-03063127211038752] Dickmanns ED Zapp A (1987) Autonomous high speed road vehicle guidance by computer vision1. IFAC Proceedings Volumes 20(5, Part 4): 221–226.

[bibr18-03063127211038752] Dittmar H (1992) The Social Psychology of Material Possessions: To Have is to Be. Hemel Hempstead: Harvester Wheatsheaf.

[bibr19-03063127211038752] Downs A (2004) Still Stuck in Traffic. Washington: Brookings Institution Press.

[bibr20-03063127211038752] *Economist* (2019) Driverless cars are stuck in a jam. The Economist, 10 October. Available at: https://www.economist.com/leaders/2019/10/10/driverless-cars-are-stuck-in-a-jam (accessed 29 October 2019).

[bibr21-03063127211038752] Edwards PN (1997) Review of Bruno Latour, Aramis, or the Love of Technology. Isis 88(2): 322–324.

[bibr22-03063127211038752] Ekbia H Nardi B (2014) Heteromation and its (dis)contents: The invisible division of labor between humans and machines. First Monday 19(6). https://firstmonday.org/article/view/5331/4090

[bibr23-03063127211038752] Elish MC (2019) Moral crumple zones: Cautionary tales in human-robot Interaction. Engaging Science, Technology and Society 5: 21.

[bibr24-03063127211038752] Eubanks V (2018) Automating Inequality: How High-Tech Tools Profile, Police, and Punish the Poor. New York, NY: St. Martin’s Press.

[bibr25-03063127211038752] Felt U Wynne B Callon M , et al. (2007) Taking European knowledge society seriously: Report of the expert group on science and governance to the Science, Economy and Society Directorate. Directorate-General for research, European Commission, Luxembourg.

[bibr26-03063127211038752] Ganesh MI (2020) The ironies of autonomy. Humanities and Social Sciences Communications 7(1): 157.

[bibr27-03063127211038752] Gieryn TF (2000) A space for place in sociology. Annual Review of Sociology 26(1): 463–496.

[bibr28-03063127211038752] Gigerenzer G (2008) Why heuristics work. Perspectives on Psychological Science 3(1): 20–29. 26158666 10.1111/j.1745-6916.2008.00058.x

[bibr29-03063127211038752] Gittleman M Monaco K (2019) Truck-driving jobs: Are they headed for rapid elimination? ILR Review 73(1): 3–24.

[bibr30-03063127211038752] Glaeser EL (2000) Cities and ethics: An essay for Jane Jacobs. Journal of Urban Affairs 22(4): 473–493.

[bibr31-03063127211038752] Goldenfein J Mulligan D Nissenbaum H (2019) Through the handoff lens: Are autonomous vehicles no-win for users. WeRobot 2019, Miami. Available at: https://robots.law.miami.edu/2019/wp-content/uploads/2019/03/Goldenfein_Through-the-Handoff-Lens.pdf (accessed 23 July 2021).

[bibr32-03063127211038752] Hennion (2017) Attachments, you say? … How a concept collectively emerges in one research group. Journal of Cultural Economy 10(1): 112–121.

[bibr33-03063127211038752] Hildebrand JM (2019) On self-driving cars as a technological sublime. Techné: Research in Philosophy and Technology 23(2): 153–173.

[bibr34-03063127211038752] Irani L (2013) The cultural work of microwork. New Media & Society 17(5): 720–739.

[bibr35-03063127211038752] JafariNaimi N (2017) Our bodies in the trolley’s path, or why self-driving cars must *not* be programmed to kill. Science, Technology, & Human Values 43(2): 302–323.

[bibr36-03063127211038752] Kaldewey D (2018) The grand challenges discourse: Transforming identity work in science and science policy. Minerva 56(2): 161–182. 29780179 10.1007/s11024-017-9332-2 PMC5948272

[bibr37-03063127211038752] Koopman P (2018) Practical experience report: Automotive safety practices vs. accepted principles. In: Gallina B Skavhaug A Bitsch F (eds) SAFECOMP 2018. Lecture Notes in Computer Science. Cham: Springer, 3–11.

[bibr38-03063127211038752] Korosec K (2015) Elon Musk says Tesla vehicles will drive themselves in two years. Fortune, 21 December. Available at: https://fortune.com/2015/12/21/elon-musk-interview/?xid=yahoo_fortune (accessed 18 November 2020).

[bibr39-03063127211038752] Krimsky S (2005) From Asilomar to industrial biotechnology: Risks, reductionism and regulation. Science as Culture 14(4): 309–323. 16619467 10.1080/09505430500368998

[bibr40-03063127211038752] Latour B (1996) Aramis, or the Love of Technology. Cambridge, MA: Harvard University Press.

[bibr41-03063127211038752] Latour B (2011) Love your Monsters. Breakthrough Journal 2(Fall): 19–26.

[bibr42-03063127211038752] Law J (1987) Technology and heterogeneous engineering: The case of Portuguese expansion. In: Bijker WE Hughes TP Pinch TJ (eds) The Social Construction of Technological Systems: New Directions in the Sociology and History of Technology. Cambridge, MA: MIT Press, 105–128.

[bibr43-03063127211038752] Law Commission and Scottish Law Commission (2019) Automated vehicles: Analysis of responses to the preliminary consultation paper. Available at: https://www.scotlawcom.gov.uk/files/2815/6093/3787/Automated_Vehicles_-_Analysis_of_Responses_to_the_Preliminary_Consultation_Paper.pdf (accessed 23 July 2021).

[bibr44-03063127211038752] Lewin K (1997) Formalization and process in psychology. In: Lewin GW (ed.) Resolving Social Conflicts; and, Field Theory in Social Science. Washington: American Psychological Association, 169–190.

[bibr45-03063127211038752] Lomasky LE (1997) Autonomy and automobility. The Independent Review 2(1): 5–28.

[bibr46-03063127211038752] Marchant G Lindor R (2012) The coming collision between autonomous vehicles and the liability system. Santa Clara Law Review 52: 1321.

[bibr47-03063127211038752] McDermid JA Jia Y Habli I (2019) Artificial intelligence safety 2019. In: Espinoza H Yu H Huang X , et al. (eds) Artificial Intelligence Safety CEUR Workshop Proceedings. 1–7.

[bibr48-03063127211038752] Merriman P (2006) ‘Mirror, signal, manoeuvre’: Assembling and governing the motorway driver in late 1950s Britain. Sociological Review 54(1): 75–92.

[bibr49-03063127211038752] Mindell DA (2015) Our Robots, Ourselves: Robotics and the Myths of Autonomy. New York, NY: Viking.

[bibr50-03063127211038752] Nader R (1973) Unsafe at Any Speed: The Designed-in Dangers of the American Automobile. London: Bantam. 10.2105/ajph.101.2.254 PMC3020193 21228290

[bibr51-03063127211038752] Norton PD (2008) Fighting Traffic: The Dawn of the Motor Age in the American City. Cambridge, MA: MIT Press.

[bibr52-03063127211038752] Nunes A Hernandez KD (2020) Autonomous taxis & public health: High cost or high opportunity cost? Transportation Research Part A: Policy and Practice 138(August): 28–36.

[bibr53-03063127211038752] Oxbotica (2020) Oxbotica: What we do. Available at: https://www.oxbotica.com/what-we-do/ (accessed 15 November 2020).

[bibr54-03063127211038752] Pfotenhauer S Laurent B Papageorgiou K , et al. (in press) Can it scale? Coming to terms with the politics of scaling in innovation and public policy. Social Studies of Science. 10.1177/03063127211048945 PMC8771885 34625011

[bibr55-03063127211038752] Piper K (2020) It’s 2020. Where are our self-driving cars? Vox, 28 February. Available at: https://www.vox.com/future-perfect/2020/2/14/21063487/self-driving-cars-autonomous-vehicles-waymo-cruise-uber (accessed 15 November 2020).

[bibr56-03063127211038752] Puig de la Bellacasa M (2010) Matters of care in technoscience: Assembling neglected things. Social Studies of Science 41(1): 85–106. 10.1177/0306312710380301 21553641

[bibr57-03063127211038752] Roughgarden T (2016) Selfish routing and the price of anarchy. In: Roughgarden T (ed.) Twenty Lectures on Algorithmic Game Theory. Cambridge: Cambridge University Press, 145–158.

[bibr58-03063127211038752] Royakkers L van Est R (2015) A literature review on new robotics: Automation from love to war. International Journal of Social Robotics 7(5): 549–570.

[bibr59-03063127211038752] SAE International (2016) Taxonomy and definitions for terms related to on-road motor vehicle automated driving systems. Available at: https://www.sae.org/standards/content/j3016_201609/ (accessed 23 July 2021).

[bibr60-03063127211038752] SAE International (2018) Taxonomy and definitions for terms related to driving automation systems for on-road motor vehicles. Available at: https://www.sae.org/standards/content/j3016_201806/ (accessed 23 July 2021).

[bibr61-03063127211038752] Seaver N (2017) Attending to the mediators. Journal of Cultural Economy 10(3): 309–313.

[bibr62-03063127211038752] Seo SA (2019) Policing the Open Road: How Cars Transformed American Freedom. Cambridge, MA: Harvard University Press.

[bibr63-03063127211038752] Sheller M (2018) Mobility Justice: The Politics of Movement in the Age of Extremes. London: Verso.

[bibr64-03063127211038752] Shladover SE (2018) Connected and automated vehicle systems: Introduction and overview. Journal of Intelligent Transportation Systems 22(3): 190–200.

[bibr65-03063127211038752] Sovacool BK Hess DJ (2017) Ordering theories: Typologies and conceptual frameworks for sociotechnical change. Social Studies of Science 47(5): 703–750. 28641502 10.1177/0306312717709363 PMC5648049

[bibr66-03063127211038752] Stayton E (2020) Humanizing autonomy: Social scientists’ and engineers’ futures for robotic cars. PhD thesis, Massachusetts Institute of Technology, MA.

[bibr67-03063127211038752] Steg L Gärling T (2007) Introduction. In: Steg L Gärling T (eds) Threats from Car Traffic to the Quality of Urban Life. Bingley: Emerald Group Publishing Limited, 1–7.

[bibr68-03063127211038752] Stilgoe J (2017) Seeing like a Tesla: How can we anticipate self-driving worlds? Glocalism 3: 1–20.

[bibr69-03063127211038752] Stilgoe J (2018) Machine learning, social learning and the governance of self-driving cars. Social Studies of Science 48(1): 25–56. 29160165 10.1177/0306312717741687

[bibr70-03063127211038752] Stilgoe J Owen R Macnaghten P (2013) Developing a framework for responsible innovation. Research Policy 42(9): 1568–1580.

[bibr71-03063127211038752] Stokes G Hallett S (1992) The role of advertising and the car. Transport Reviews 12(2): 171–183.

[bibr72-03063127211038752] Suchman L Weber J (2016) Human–machine autonomies. In: Bhuta N Beck S Geiβ R , et al. (eds) Autonomous Weapons Systems: Law, Ethics, Policy. Cambridge: Cambridge University Press, 75–102.

[bibr73-03063127211038752] Taylor BD (2002) Rethinking traffic congestion. Access Magazine 1(21): 8–16.

[bibr74-03063127211038752] Tennant C Howard S Stares S (in press) Building the UK vision of a driverless future: A public inquiry case study. Humanities and Social Sciences Communications.

[bibr75-03063127211038752] Tennant C Stares S Howard S (2019) Public discomfort at the prospect of autonomous vehicles: Building on previous surveys to measure attitudes in 11 countries. Transportation Research Part F: Traffic Psychology and Behaviour 64: 98–118.

[bibr76-03063127211038752] Tesla (2019) Tesla autonomy day. Available at: https://www.youtube.com/watch?v=Ucp0TTmvqOE (accessed 29 July 2020).

[bibr77-03063127211038752] Thrun S (2011a) Google’s driverless car. Available at: https://www.ted.com/talks/sebastian_thrun_google_s_driverless_car (accessed 7 November 2020).

[bibr78-03063127211038752] Thrun S (2011b) Leave the driving to the car, and reap benefits in safety and mobility. New York Times, 5 December. Available at: https://www.nytimes.com/2011/12/06/science/sebastian-thrun-self-driving-cars-can-save-lives-and-parking-spaces.html (accessed November 7, 2020).

[bibr79-03063127211038752] Tibken S (2018) Waymo CEO: Autonomous cars won’t ever be able to drive in all conditions. CNET, 13 November. Available at: https://www.cnet.com/news/alphabet-google-waymo-ceo-john-krafcik-autonomous-cars-wont-ever-be-able-to-drive-in-all-conditions/ (accessed 30 May 2020).

[bibr80-03063127211038752] Townsend A (2020) Ghost Road: Beyond the Driverless Car. New York: WW Norton & Company.

[bibr81-03063127211038752] Urry J (2004) The ‘system’ of automobility. Theory, Culture & Society 21(4–5): 25–39.

[bibr82-03063127211038752] van der Vecht B van Diggelen J Peeters M , et al. (2018) SAIL: A social artificial intelligence layer for human-machine teaming. In: Advances in Practical Applications of Agents, Multi-agent Systems, and Complexity: The PAAMS Collection - 16th International Conference, Toledo, Spain. Cham: Springer, 262–274.

[bibr83-03063127211038752] van Lente H (2000) Forceful futures: From promise to requirement. In: Webster A Brown N Rappert B (eds) Contested Futures: A Sociology of Prospective Techno-Science. Aldershot: Ashgate, 43–64.

[bibr84-03063127211038752] van Lente H Bakker S (2010) Competing expectations: The case of hydrogen storage technologies. Technology Analysis & Strategic Management 22(6): 693–709.

[bibr85-03063127211038752] Vinsel L (2019) Moving Violations: Automobiles, Experts, and Regulations in the United States. Baltimore, MD: John Hopkins University Press.

[bibr86-03063127211038752] Vonnegut K (1952/1985) Player Piano. London: Panther Books.

[bibr87-03063127211038752] Waymo (2020) Let’s talk self-driving. Available at: https://www.letstalkselfdriving.com/about/waymo-zones.html (accessed 30 May 2020).

[bibr88-03063127211038752] Waymo (2021) Let’s talk autonomous driving. Available at: https://www.ltad.com/about/waymo-zones.html (accessed 23 July 2021)

[bibr89-03063127211038752] Waymo Team (2019) Waymo @ IAA Frankfurt 2019. Medium, 12 September. Available at: https://medium.com/waymo/waymo-iaa-frankfurt-2019-b3cca36d8479 (accessed 13 March 2020).

[bibr90-03063127211038752] Wetmore JM (2020) Überlegungen zum Traum vom automatisierten Fahrzeug. Visionen selbsttätigen Fahrens aus den letzten 80 Jahren [Reﬂecting on the dream of automated vehicles: Visions of hands free driving over the past 80 years]. TG Technikgeschichte 87(1): 69–94.

[bibr91-03063127211038752] Winfield AFT Jirotka M (2018) Ethical governance is essential to building trust in robotics and artificial intelligence systems. Philosophical Transactions of the Royal Society A Math, Physics and Engineering Science 376, 20180085. 10.1098/rsta.2018.0085 PMC6191667 30323000

[bibr92-03063127211038752] Winner L (1977) Autonomous Technology: Technics-out-of-Control as a Theme in Political Thought. Cambridge, MA: MIT Press.

[bibr93-03063127211038752] Zworykin V Heyer M (1975) Oral-History:Vladimir Zworykin. Available at: https://ethw.org/Oral-History:Vladimir_Zworykin (accessed 15 November 2020).

